# Epidemiology of hospitalized strongyloidiasis in Japan: A nationwide administrative database study

**DOI:** 10.1016/j.parepi.2026.e00555

**Published:** 2026-10-10

**Authors:** Ryo Torii, Naoaki Ohkubo, Kei Yamasaki, Masahiro Tahara, Kei Nakamura, Kentaro Akata, Hiroaki Ikegami, Chinatsu Nishida, Makoto Okawara, Yoshihisa Fujino, Shinya Matsuda, Kiyohide Fushimi, Hiroshi Mukae, Kazuhiro Yatera

**Affiliations:** a Department of Respiratory Medicine, Wakamatsu Hospital of the University of Occupational and Environmental Health, Japan, Kitakyushu, Japan; b Department of Environmental Epidemiology, Institute of Industrial Ecological Sciences, University of Occupational and Environmental Health, Japan, Kitakyushu, Japan; c Department of Respiratory Medicine, University of Occupational and Environmental Health, Japan, Kitakyushu, Japan; d Division of Infection Control and Prevention, University of Occupational and Environmental Health, Japan, Kitakyushu, Japan; e Department of Environmental Health Engineering, Institute of Industrial Ecological Sciences, University of Occupational and Environmental Health, Japan, Kitakyushu, Japan; f Department of Preventive Medicine and Community Health, University of Occupational and Environmental Health, Japan, Kitakyushu, Japan; g Department of Nursing, Fukuoka International University of Health and Welfare, Fukuoka, Japan; h Department of Health Policy and Informatics, Institute of Science Tokyo, Tokyo, Japan; i Department of Respiratory Medicine, Nagasaki University Graduate School of Biomedical Sciences, Nagasaki, Japan

**Keywords:** Administrative database, In-hospital mortality, Diagnosis procedure combination, Disseminated strongyloidiasis, Nationwide study, Strongyloidiasis

## Abstract

Strongyloidiasis remains endemic in parts of Japan, particularly Okinawa Prefecture, and can progress to disseminated disease with high mortality; however, nationwide data on hospitalized patients, including regional differences and the impact of disseminated disease, are limited. We conducted a retrospective nationwide study using the Diagnosis Procedure Combination database to identify hospitalized patients with strongyloidiasis in Japan between fiscal years 2014 and 2020, using ICD-10 code B78, with disseminated cases defined as B78.7. Demographic characteristics, geographic distribution, and outcomes were analyzed, with a focus on disseminated disease and regional differences between Okinawa and other areas. After excluding duplicate cases, 252 hospitalized patients were included (mean age, 77.6 years; male-to-female ratio, 1.47). Most patients were from the Kyushu and Okinawa regions, with Okinawa accounting for 82.1% of cases. Disseminated strongyloidiasis was identified in 44 patients (17.5%) and was associated with poorer neurological status at admission and higher in-hospital mortality; 11 patients with disseminated disease died during hospitalization (25.0%). Severe complications, including sepsis (54.5%) and meningitis (25.0%), were frequently observed in disseminated cases. Patients hospitalized outside Okinawa were younger than those hospitalized in Okinawa, whereas the proportions of disseminated disease and in-hospital mortality did not differ significantly between regions. In Japan, hospitalized patients diagnosed with strongyloidiasis were predominantly concentrated in Okinawa; however, severe cases also occurred in other regions. These findings highlight the importance of considering strongyloidiasis in patients with relevant epidemiological backgrounds, particularly in the setting of immunosuppression, regardless of their current geographic location.

## Graphical abstract

**Figure:**
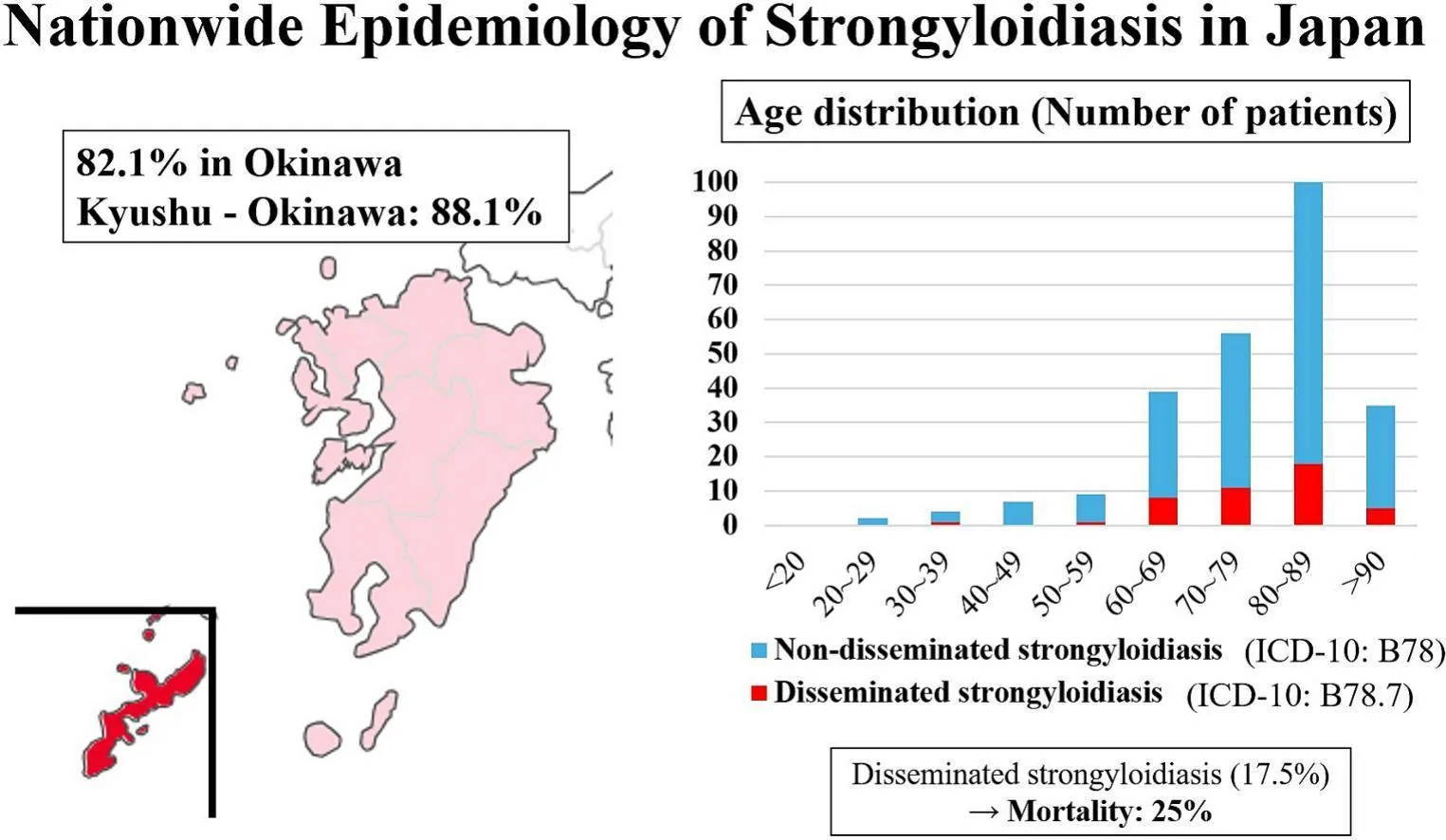

## 1 Introduction

Strongyloidiasis is a parasitic infection caused by *Strongyloides stercoralis* and is estimated to affect tens of millions of people worldwide (Nutman, 2017). It is widely recognized as a neglected tropical disease, reflecting its global burden and the challenges associated with its diagnosis and control (Nutman, 2017). Infection typically occurs through percutaneous exposure to soil contaminated with infective filariform larvae, and the unique ability of *S. stercoralis* to cause autoinfection allows the parasite to persist for decades within a human host (Nutman, 2017; Schär et al., 2013).

In immunocompetent individuals, strongyloidiasis often manifests as an asymptomatic or mildly symptomatic chronic infection (Nutman, 2017). In contrast, immunosuppressed patients may develop hyperinfection syndrome or disseminated strongyloidiasis, conditions associated with severe bacterial infections and high mortality (Mejia and Nutman, 2012). Because these life-threatening manifestations may be triggered by corticosteroid therapy or other immunosuppressive treatments, delayed or missed diagnosis of strongyloidiasis represents a clinically important problem in internal medicine practice.

In Japan, strongyloidiasis has been considered endemic in the southwestern islands, including Okinawa and the Amami Islands, as demonstrated by previous epidemiological studies (Ikuno et al., 2020), while sporadic cases have also been reported in other regions, including large metropolitan areas of Honshu (Okumura et al., 2026). Although the incidence of soil-transmitted helminthiases declined markedly following mass screening programs, improvements in sanitation, and regulatory changes limiting the use of human feces as fertilizer (Kobayashi et al., 2006), strongyloidiasis has not been eradicated in Japan. This persistence is thought to be attributable to the parasite's autoinfective life cycle, which enables long-term infection even in the absence of ongoing environmental exposure (Nutman, 2017). Recent studies have reported declining seroprevalence in Okinawa, suggesting changes in the epidemiology of strongyloidiasis in Japan (Kosuge et al., 2026).

Epidemiological data on strongyloidiasis in Japan have mainly been derived from laboratory-based surveillance and case reports. For example, a nationwide laboratory survey identified 279 cases over an 18-year period and described their demographic and geographic characteristics (Ikuno et al., 2020); however, data focusing on hospitalized patients, disease severity, and regional distribution remain limited. In particular, the incidence and geographic patterns of severe or disseminated strongyloidiasis have not been well characterized at the national level.

The Diagnosis Procedure Combination (DPC) database is a nationwide administrative inpatient database that captures diagnoses and treatments recorded during acute-care hospitalizations in Japan (Hayashida et al., 2021; Matsuda, 2016). Using this database, we conducted a retrospective nationwide study with three specific objectives: (1) to characterize the demographic and geographic distribution and temporal trends of hospitalized patients with strongyloidiasis in Japan; (2) to describe the characteristics and in-hospital outcomes of patients with disseminated strongyloidiasis; and (3) to compare the characteristics and outcomes of patients hospitalized in Okinawa Prefecture with those hospitalized in other regions of Japan.

## 2 Materials and methods

### 2.1 Participants and the source database

Most acute care hospitals in Japan submit DPC data to the Ministry of Health, Labor and Welfare. We collected these data through the DPC Research Institute and used them in this analysis. Data were extracted for fiscal years (FYs) 2014 through 2020. Because the Japanese fiscal year runs from April to March of the following year, the study period corresponded to April 2014 through March 2021.

The DPC system classifies hospitalized patients according to their principal diagnoses and the treatments administered during hospitalization. The DPC system is a case-mix–based reimbursement system implemented by the Ministry of Health, Labour and Welfare of Japan and widely adopted by acute-care hospitals nationwide (Hayashida et al., 2021; Matsuda, 2016). The system classifies hospitalized patients according to their diagnoses and procedures, and reimbursement is determined based on a combination of per-diem payments and fee-for-service components (Hayashida et al., 2021). The database contains information on primary diagnoses, comorbidities documented at admission, and conditions arising during hospitalization, as well as demographic variables, including age, sex, residential area, physical characteristics, length of hospital stay, discharge status, and in-hospital complications (Hayashida et al., 2021). Diagnoses are recorded in Japanese and coded using the International Classification of Diseases, 10th Revision (ICD-10) (Matsuda, 2016).

Patients eligible for inclusion were those hospitalized during the study period with strongyloidiasis recorded at admission using the ICD-10 code B78. Both primary and secondary diagnoses recorded at admission were included because the overall cohort analysis was intended to characterize the nationwide epidemiological and geographic distribution of hospitalized patients in whom strongyloidiasis was recorded at admission, irrespective of whether strongyloidiasis was the principal reason for hospitalization. Repeated hospitalizations considered to represent the same patient were excluded to avoid duplicate counting. Disseminated strongyloidiasis was identified using the ICD-10 code B78.7. No age-based inclusion or exclusion criterion was applied. For the analysis of age distribution, patients were categorized into 10-year age groups (<20, 20–29, 30–39, 40–49, 50–59, 60–69, 70–79, 80–89, and ≥ 90 years). We extracted the following variables: age, sex, residential area, Japan Coma Scale (JCS) score, length of hospital stay, discharge status, presence of disseminated strongyloidiasis, in-hospital complications (pneumonia, meningitis, and sepsis), comorbidities including diabetes mellitus, hypertension, cerebrovascular disease, chronic heart failure, ischemic heart disease, and human T-cell leukemia virus type 1 (HTLV-1) infection or carrier status, and antiparasitic treatment, including ivermectin and albendazole administration and treatment duration. The JCS is a widely used scale for assessing the level of consciousness in Japan. A JCS score of 0 indicates alert consciousness, scores of 1–3 indicate that the patient is awake without stimulation, scores of 10–30 indicate that the patient is arousable with stimulation, and scores of 100–300 indicate that the patient is unarousable with stimulation (Nakajima et al., 2023). In the present study, JCS scores were categorized into three groups (0–3, 10–30, and 100–300) and were used as an indicator of neurological status at admission. In addition, patients were categorized into those from Okinawa Prefecture and those from other regions of Japan, and their characteristics were compared.

### 2.2 Statistical analysis

Patient characteristics were summarized using descriptive statistics. Continuous variables are presented as mean (standard deviation [SD]) or median (minimum–maximum), as appropriate according to data distribution. Continuous variables were compared using the Student's *t*-test or the Mann–Whitney *U* test, while categorical variables were analyzed using the chi-square test.

All statistical analyses were performed using SPSS software (version 27; IBM Corp., Armonk, NY, USA). A two-sided *P* value of less than 0.05 was considered statistically significant. Comparisons between patients with and without disseminated strongyloidiasis were exploratory and descriptive and were not intended to identify independent predictors or establish causal associations.

### 2.3 Ethical considerations

This study was approved by the Institutional Review Board of our institution (approval number: R4–045). The requirement for informed consent was waived owing to the retrospective nature of the study and the use of anonymized administrative data.

## 3 Results

### 3.1 Patient characteristics

Between fiscal years 2014 and 2020, 262 patients were diagnosed with strongyloidiasis at the time of hospital admission. After excluding duplicate cases, 252 hospitalized patients were included in the final analysis. The study population comprised 150 men and 102 women, with a mean age of 77.6 years. Baseline characteristics at admission are summarized in Table 1. The male-to-female ratio was 1.47, indicating a male predominance. The most common comorbidity was hypertension (32.5%), followed by diabetes mellitus (17.1%) and cerebrovascular disease (17.1%). Among the 252 patients, 152 (60.3%) received ivermectin therapy, with a median treatment duration of 2 days. No patients underwent surgical procedures related to strongyloidiasis during hospitalization.

**Table 1:** Baseline characteristics and clinical outcomes of hospitalized patients with strongyloidiasis.

Characteristics	Disseminated strongyloidiasis (*n* = 44)	Non-disseminated strongyloidiasis (*n* = 208)	Total (*n* = 252)	*P* value
Demographic characteristics				
Age, years, mean (SD)	78.2 (11.9)	77.5 (14.1)	77.6 (13.7)	0.72
Male sex, n (%)	26 (59.1%)	124 (59.6%)	150 (59.5%)	0.95
Neurological status at admission				
Japan Coma Scale (JCS)				<0.001
0–3	28 (63.6%)	193 (92.8%)	221 (87.7%)	
10–30	10 (22.7%)	12 (5.8%)	22 (8.7%)	
100–300	6 (13.6%)	3 (1.4%)	9 (3.6%)	
Comorbidities at admission				
Hypertension	14 (31.8%)	68 (32.7%)	82 (32.5%)	0.91
Diabetes mellitus	8 (18.2%)	35 (16.8%)	43 (17.1%)	0.82
Cerebrovascular disease	7 (15.9%)	36 (17.3%)	43 (17.1%)	0.83
Heart failure	5 (11.4%)	26 (12.5%)	31 (12.3%)	0.84
Ischemic heart disease	2 (4.5%)	22 (10.6%)	24 (9.5%)	0.22
HTLV-1 carrier or infection	4 (9.1%)	12 (5.8%)	16 (6.3%)	0.42
Major complications during hospitalization				
Pneumonia	15 (34.1%)	61 (29.3%)	76 (30.2%)	0.53
Sepsis	24 (54.5%)	23 (11.1%)	47 (18.7%)	<0.001
Meningitis	11 (25.0%)	9 (4.3%)	20 (7.9%)	<0.001
Treatment				
Ivermectin	31 (70.5%)	121 (58.2%)	152 (60.3%)	0.140
Duration of ivermectin treatment, days	3 (1−23)	2 (1–14)	2 (1–23)	<0.001
Albendazole	1 (2.3%)	0	1 (0.4%)	–
Duration of albendazole treatment, days	3	0	3	–
Clinical outcomes				
Length of hospital stay, days, mean (SD)	39.7 (42.8)	27.2 (33.3)	29.3 (35.4)	0.003
In-hospital mortality	11 (25.0%)	12 (5.8%)	23 (9.1%)	<0.001

Data are presented as mean (SD), median (minimum–maximum), or number (%).

Disseminated strongyloidiasis, which is associated with a high risk of severe disease, was diagnosed in 44 of the 252 hospitalized patients (17.5%). Compared with patients without disseminated disease, those with disseminated strongyloidiasis had significantly worse neurological status at admission, as reflected by higher Japan Coma Scale scores, and a significantly higher in-hospital mortality rate. The mean length of hospital stay was also significantly longer in patients with disseminated disease than in those without disseminated disease (39.7 vs. 27.2 days, *P* = 0.003). Severe complications were frequently observed in disseminated cases, including sepsis (54.5%) and meningitis (25.0%). HTLV-1 infection or carrier status was recorded in 4 of 44 patients (9.1%) with disseminated strongyloidiasis and 12 of 208 patients (5.8%) without disseminated disease, although the difference was not statistically significant (*P* = 0.42). No significant differences were observed in mean age or sex distribution between patients with and without disseminated strongyloidiasis.

### 3.2 Geographic distribution and temporal trends

Fig. 1 illustrates the geographic distribution of hospitalized patients with strongyloidiasis by region and prefecture in Japan. The majority of patients were from the Kyushu and Okinawa regions, accounting for 88.1% of all cases. At the prefectural level, Okinawa Prefecture accounted for the largest proportion of cases (82.1%), followed by Kagoshima Prefecture (3.6%). The mean annual number of hospitalized patients with strongyloidiasis was 36 during the seven-year study period, showing a gradual decreasing trend over time (Fig. 2). The annual number of hospitalized patients decreased from 52 in fiscal year 2014 to 20 in fiscal year 2020, whereas disseminated cases ranged from 3 to 10 per year without a clear decreasing trend. Detailed annual numbers of overall, disseminated, and non-disseminated strongyloidiasis cases are presented in Supplementary Table S1. The age distribution of the overall study population is shown in Fig. 3; the number of hospitalized patients increased with advancing age, peaking in the 80–89-year age group (*n* = 100). Similarly, the age distribution of disseminated strongyloidiasis peaked in the 80–89-year age group (*n* = 18). Most hospitalized patients were older adults, and both overall and disseminated cases were concentrated in the older age groups. Detailed age-stratified numbers of overall, disseminated, and non-disseminated strongyloidiasis cases are presented in Supplementary Table S2. No patients younger than 20 years were identified during the study period.

### 3.3 Comparison between Okinawa and non-Okinawa regions

Characteristics of hospitalized patients with strongyloidiasis in Okinawa Prefecture and those in other regions are presented in Table 2. Patients from Okinawa Prefecture were significantly older, with a mean age of 79.6 years, whereas those from outside Okinawa were younger, with a mean age of 68.2 years. Patients hospitalized outside Okinawa were more likely to receive antiparasitic treatment, whereas the proportion of disseminated strongyloidiasis and in-hospital mortality did not differ significantly between regions.

**Fig. 1:**
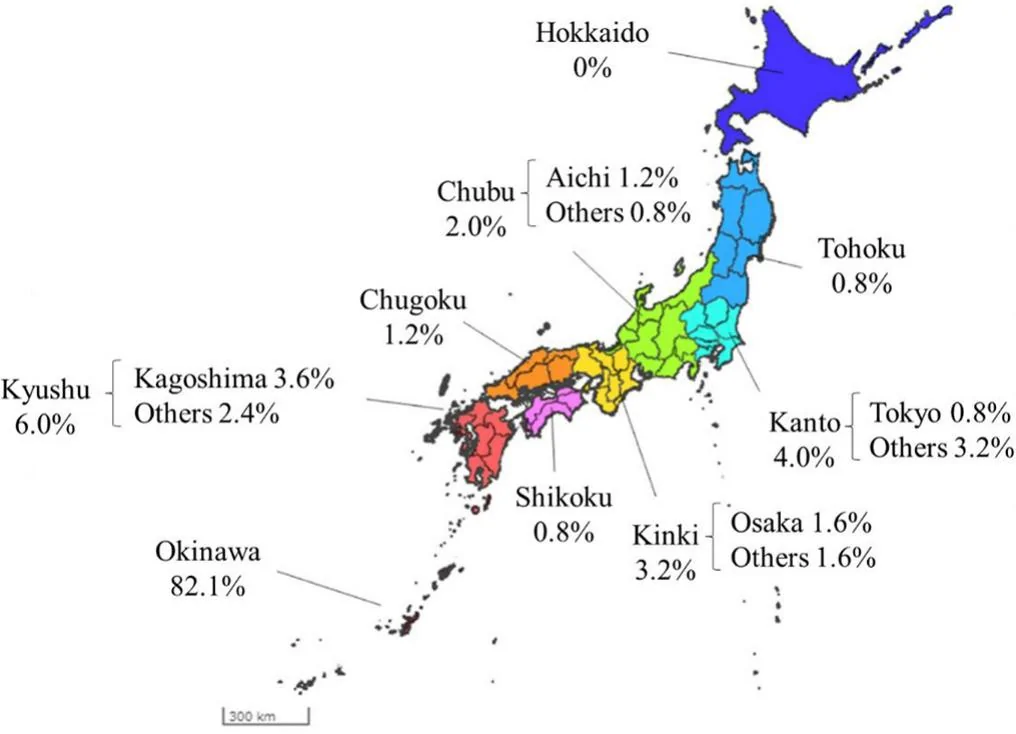
Geographic distribution of hospitalized patients with strongyloidiasis in Japan. Geographic distribution of hospitalized patients diagnosed with strongyloidiasis across Japan between fiscal years 2014 and 2020. The number of patients is shown by prefecture based on the Diagnosis Procedure Combination database. Most patients were concentrated in the Kyushu and Okinawa regions, particularly in Okinawa Prefecture.

**Fig. 2:**
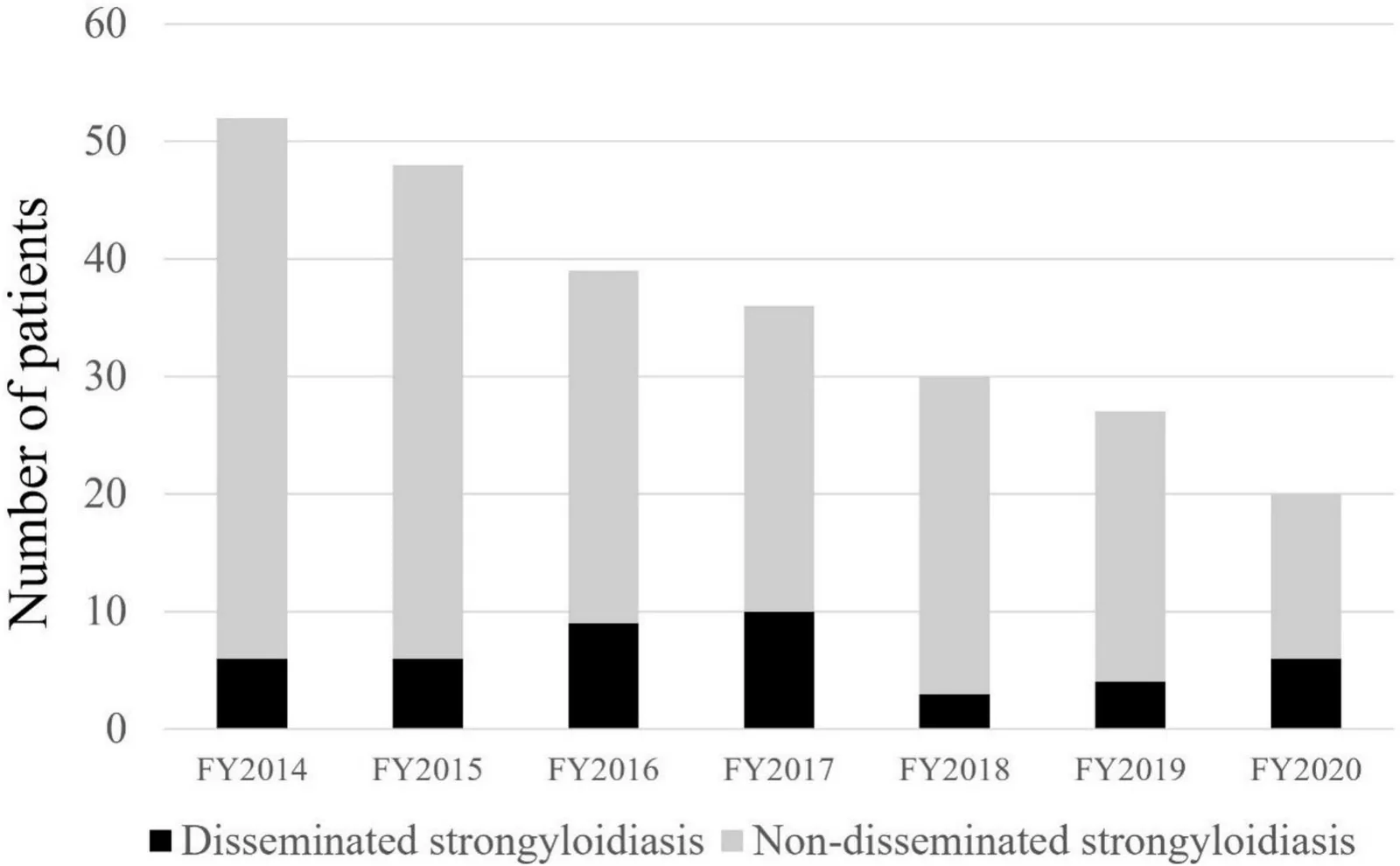
Annual trends in hospitalized patients with strongyloidiasis in Japan. Annual trends in the number of hospitalized patients with strongyloidiasis in Japan between fiscal years 2014 and 2020. The numbers of patients with disseminated strongyloidiasis and those with non-disseminated strongyloidiasis are shown separately. While the total number of hospitalized patients gradually decreased over the study period, the number of patients with disseminated strongyloidiasis remained relatively stable.

**Fig. 3:**
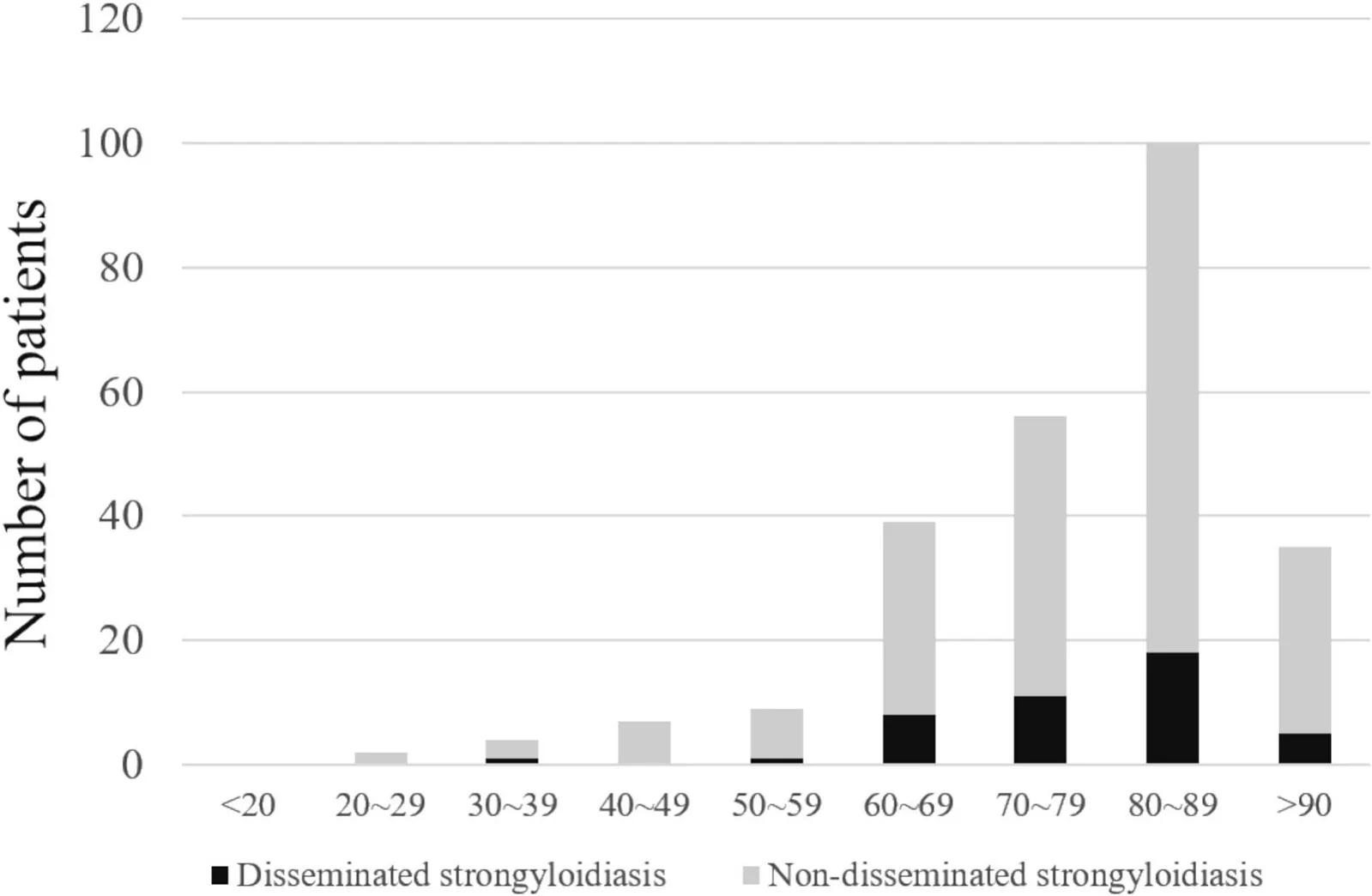
Age distribution of hospitalized patients with strongyloidiasis. Age distribution of hospitalized patients with strongyloidiasis in Japan between fiscal years 2014 and 2020. Patients are stratified by disseminated and non-disseminated strongyloidiasis. The number of patients increased with advancing age, with a peak in the 80–89-year age group in both groups. No patients younger than 20 years were hospitalized during the study period.

**Table 2:** Comparison of clinical characteristics of hospitalized patients with strongyloidiasis between Okinawa Prefecture and other regions of Japan.

Characteristics	Okinawa (*n* = 207)	Other regions (*n* = 45)	Total (*n* = 252)	P value
Demographic characteristics				
Age, years, mean (SD)	79.6 (11.9)	68.2 (17.5)	77.6 (13.7)	<0.001
Male sex, n (%)	123 (59.4%)	27 (60.0%)	150 (59.5%)	0.94
Disease severity and treatment				
Disseminated strongyloidiasis	34 (16.4%)	10 (22.2%)	44 (17.5%)	0.36
Ivermectin	115 (55.6%)	37 (82.2%)	152 (60.3%)	0.002
Duration of ivermectin treatment, days	2 (1–23)	2 (1–18)	2 (1–23)	0.44
Clinical outcomes				
Length of hospital stay, days, mean (SD)	28.0 (36.4)	35.1 (43.9)	29.3 (35.4)	0.081
In-hospital mortality	18 (8.7%)	5 (11.1%)	23 (9.1%)	0.59

Data are presented as mean (SD), median (minimum–maximum), or number (%).

## 4 Discussion

In this nationwide inpatient study, we demonstrated that disseminated strongyloidiasis remains a significant cause of severe infection and in-hospital mortality in Japan. Although strongyloidiasis is often regarded as a chronic parasitic infection, our findings highlight its potential to present as a life-threatening infectious disease characterized by sepsis and meningitis. Notably, differences in age and treatment patterns were observed between patients hospitalized in Okinawa Prefecture and those in other regions, whereas the proportions of disseminated disease and in-hospital mortality did not differ significantly between regions.

Previous reviews of reported cases between 2000 and 2013 have shown that the majority of strongyloidiasis cases in Japan occurred in the southwestern islands, including Okinawa and Amami (Yoshikawa et al., 2014). In addition, a nationwide laboratory-based survey using stool samples collected between 2000 and 2017 demonstrated that most cases were identified in southern Japan, with Okinawa Prefecture accounting for the largest proportion (65.2%), followed by Kagoshima Prefecture (7.2%) and Tokyo (7.2%) (Ikuno et al., 2020). Consistent with these earlier reports, our nationwide inpatient analysis showed that hospitalized patients with strongyloidiasis were predominantly distributed in the Kyushu and Okinawa regions. At the prefectural level, Okinawa Prefecture accounted for the majority of cases (82.1%), followed by Kagoshima Prefecture (3.6%), further supporting the persistent geographic clustering of this infection in southwestern Japan. It should be noted that the geographic distribution in this study reflects the location of hospitalization rather than the site of infection.

Laboratory-based surveillance data have suggested that the annual number of strongyloidiasis cases in Japan remained relatively stable, with an average of at least 15.5 cases per year between 2000 and 2017 (Ikuno et al., 2020). In contrast, although our study covered a shorter observation period of seven years from FY2014 to FY2020, the mean annual number of hospitalized patients diagnosed with strongyloidiasis was 36, and a gradual decreasing trend was observed over time. Differences in data sources, sampling methods, and study periods may partly explain this discrepancy. Nevertheless, given that a certain number of hospitalized cases continued to be identified throughout the study period, strongyloidiasis remains a clinically relevant condition that warrants ongoing attention in Japan.

In the present study, strongyloidiasis predominantly affected elderly individuals, with a clear male predominance. The male-to-female ratio was 1.47, the mean patient age was 77.6 years, and the highest number of cases was observed in the 80–89-year age group. These demographic characteristics are consistent with those reported in a nationwide laboratory-based survey conducted in Japan between 2000 and 2017, which showed a male-to-female ratio of 2.1 and a peak incidence in patients aged 80–89 years (Ikuno et al., 2020). Several factors may contribute to the observed male predominance. Historically, individuals in endemic regions—particularly men—were more likely to walk barefoot during agricultural work, increasing opportunities for percutaneous infection (Mukaigawara et al., 2020). In addition, experimental and clinical studies have suggested higher susceptibility to infection (Dawkins et al., 1980) and reduced responsiveness to anthelmintic treatment in men (Kobayashi et al., 1996), which may partly explain the male predominance observed in this study. The extremely low incidence among younger individuals likely reflects a marked reduction in opportunities for new environmental exposure in recent decades, supporting the notion that most current cases represent long-standing chronic infections acquired in the past (Mejia and Nutman, 2012).

Disseminated strongyloidiasis is a severe and potentially life-threatening manifestation of strongyloidiasis. In the present study, disseminated strongyloidiasis was identified in 44 of 252 hospitalized patients and was associated with poorer neurological status at admission, as reflected by higher Japan Coma Scale scores, as well as a higher in-hospital mortality rate compared with non-disseminated disease, whereas no significant differences were observed in mean age or sex distribution. Previous studies have reported mortality rates exceeding 85% for disseminated strongyloidiasis outside Japan (Mejia and Nutman, 2012; Boggild et al., 2016), while studies conducted in Japan have reported lower but still substantial mortality rates ranging from 11.5% to 56.3% (Mukaigawara et al., 2020). In our study, 11 of the 44 patients with disseminated strongyloidiasis died during hospitalization, corresponding to a mortality rate of 25.0%, which is broadly comparable to previous Japanese reports. Consistent with earlier findings (Pukkila-Worley et al., 2014), severe complications such as sepsis (54.5%) and meningitis (25.0%) were more frequent in patients with disseminated strongyloidiasis and were likely to have contributed to mortality.

Immunosuppression is a well-established risk factor for the development of hyperinfection and disseminated strongyloidiasis (Okumura et al., 2026; Boggild et al., 2016). Strongyloidiasis is characterized by an autoinfective life cycle that allows *Strongyloides stercoralis* to persist within the human host for decades (Boggild et al., 2016). Under immunosuppressive conditions, such as corticosteroid or other immunosuppressive therapy, HTLV-1 infection, hematologic malignancies, and solid organ or hematopoietic stem cell transplantation, this autoinfection cycle can be markedly accelerated, leading to hyperinfection or dissemination (Mejia and Nutman, 2012). In the present study, HTLV-1 infection or carrier status was numerically more common among patients with disseminated strongyloidiasis than among those with non-disseminated disease, although the difference was not statistically significant. In addition, patients with disseminated strongyloidiasis frequently developed severe secondary infections, including sepsis and meningitis, which were strongly associated with in-hospital mortality. These findings are consistent with the concept that impaired host immunity facilitates accelerated autoinfection and subsequent bacterial translocation, contributing to fatal outcomes in disseminated strongyloidiasis (Pukkila-Worley et al., 2014).

We compared the characteristics of hospitalized patients with strongyloidiasis in Okinawa Prefecture and those in other regions of Japan (Table 2). To our knowledge, no previous studies have directly compared patients from Okinawa and other areas in Japan. Although sex distribution did not differ, patients hospitalized outside Okinawa were younger than those from Okinawa (mean age, 68.2 vs. 79.6 years). The proportions of disseminated strongyloidiasis and in-hospital mortality were numerically higher among patients hospitalized outside Okinawa, although these differences were not statistically significant. One possible explanation is that some patients hospitalized outside Okinawa may have acquired *S. stercoralis* infection years or decades earlier while living in domestic endemic areas, such as Okinawa or the Amami Islands, and subsequently relocated to other regions of Japan. Similarly, some patients may have acquired the infection while residing in endemic regions outside Japan. Because the autoinfective life cycle of *S. stercoralis* allows infection to persist for decades (Nutman, 2017; Schär et al., 2013), hyperinfection or disseminated disease may develop long after the initial infection, particularly following immunosuppression. Indeed, disseminated strongyloidiasis following chemoradiotherapy for lung cancer has been reported in a patient living in a nonendemic area of Japan (Okumura et al., 2026). Thus, the geographic location at the time of hospitalization does not necessarily reflect the site where infection was originally acquired, and a history of residence in endemic areas may be relevant when evaluating patients at risk of severe strongyloidiasis. The older age of patients hospitalized in Okinawa may similarly reflect the long-term persistence of chronic strongyloidiasis acquired in the past, as previous studies have reported a high prevalence of long-standing infection among older individuals in endemic areas (Hirata et al., 2001). Taken together, these findings emphasize the importance of considering strongyloidiasis in patients with relevant epidemiological backgrounds, particularly when immunosuppressive therapy is planned or administered, regardless of their current geographic location.

This study has several limitations. First, the DPC database lacks detailed clinical information, such as parasitological and laboratory findings, imaging results, and disease severity; therefore, diagnoses were based on administrative records reflecting the clinical judgments of the treating physicians, without microbiological confirmation. Consequently, some cases may have included suspected or misclassified strongyloidiasis. Moreover, the diagnostic accuracy of the ICD-10 codes used to identify strongyloidiasis and disseminated strongyloidiasis has not been clinically validated in the present DPC dataset. In addition, the ICD-10 code B78.7 used to identify disseminated strongyloidiasis may not fully distinguish hyperinfection syndrome from disseminated disease. Furthermore, because patients with strongyloidiasis recorded as either a primary or secondary diagnosis were included, some hospitalizations may have been primarily attributable to conditions other than strongyloidiasis. Therefore, clinical outcomes in the overall cohort cannot necessarily be attributed directly to strongyloidiasis. Second, because the DPC database includes inpatient data only, patients managed exclusively in outpatient settings were not captured, potentially resulting in an overrepresentation of severe cases, including disseminated strongyloidiasis. Third, although repeated hospitalizations were excluded using patients' sex and date of birth, complete elimination of duplicate cases cannot be guaranteed. Finally, information on exposure history, route of infection, birthplace, nationality, lifetime residential history, previous residence in endemic areas, and detailed immunological status was unavailable. Therefore, the geographic location where *S. stercoralis* infection was originally acquired could not be determined.

## 5 Conclusion

In this nationwide inpatient study using the DPC database, hospitalized patients diagnosed with strongyloidiasis were predominantly concentrated in Okinawa Prefecture, but severe cases, including disseminated strongyloidiasis, were also observed in other regions. Disseminated strongyloidiasis accounted for a substantial proportion of hospitalized cases and was associated with high in-hospital mortality and severe complications such as sepsis and meningitis. Because *S. stercoralis* infection can persist for decades after acquisition, the geographic location at the time of hospitalization may not reflect the original site of infection. Physicians should therefore consider strongyloidiasis in patients with relevant epidemiological backgrounds, particularly when immunosuppressive therapy is planned or administered, regardless of their current geographic location.

The following are the supplementary data related to this article.

Supplementary Table S1 Annual numbers of hospitalized patients with strongyloidiasis according to disease classification.

Supplementary Table S2 Age distribution of hospitalized patients with strongyloidiasis according to disease classification.

## CRediT authorship contribution statement

**Ryo Torii:** Writing – original draft, Visualization, Data curation. **Naoaki Ohkubo:** Writing – review & editing, Data curation. **Kei Yamasaki:** Writing – review & editing, Writing – original draft, Visualization, Supervision, Methodology, Formal analysis, Data curation. **Masahiro Tahara:** Writing – review & editing. **Kei Nakamura:** Writing – review & editing. **Kentaro Akata:** Writing – review & editing. **Hiroaki Ikegami:** Writing – review & editing. **Chinatsu Nishida:** Writing – review & editing. **Makoto Okawara:** Writing – review & editing, Data curation. **Yoshihisa Fujino:** Writing – review & editing, Data curation. **Shinya Matsuda:** Writing – review & editing. **Kiyohide Fushimi:** Writing – review & editing. **Hiroshi Mukae:** Writing – review & editing. **Kazuhiro Yatera:** Writing – review & editing, Supervision.

## Ethics approval

This study was approved by the Institutional Review Board of the University of Occupational and Environmental Health, Japan (approval number: R4-045). The requirement for informed consent was waived owing to the retrospective nature of the study and the use of anonymized administrative data.

## Funding

This research did not receive any specific grant from funding agencies in the public, commercial, or not-for-profit sectors.

## Declaration of competing interest

The authors declare that they have no known competing financial interests or personal relationships that could have appeared to influence the work reported in this paper.
